# Liver fibrosis is closely linked with metabolic-associated diseases in patients with autoimmune hepatitis

**DOI:** 10.1007/s12072-024-10727-w

**Published:** 2024-09-09

**Authors:** Kehui Liu, Mingyang Feng, Wanqing Chi, Zhujun Cao, Xiaoyin Wang, Yezhou Ding, Gangde Zhao, Ziqiang Li, Lanyi Lin, Shisan Bao, Hui Wang

**Affiliations:** 1 Department of Infectious Diseases, Ruijin Hospital, Shanghai Jiao Tong University School of Medicine, Shanghai, 200025 China; 2 Epidemiology of Microbial Disease, Yale University School of Public Health, New Haven, USA

**Keywords:** MADs, NALFD, AIH, Cirrhosis

## Abstract

**Background:**

This cross-sectional study aimed to investigate the impact of metabolic-associated diseases (MADs) on patients with autoimmune hepatitis (AIH).

**Methods:**

The study analyzed the clinical characteristics of 283 AIH patients who underwent liver biopsy between January 2016 and February 2022 in Ruijin Hospital, Shanghai, China.

**Results:**

Among the identified AIH patients (*n* = 283), 87.3%, 23.0%, or 43.1% had MADs, non-alcoholic fatty liver disease (NAFLD), or severe fibrosis, respectively. The proportion of diabetes mellitus (DM) was significantly higher in patients with severe liver fibrosis than in those with mild or moderate fibrosis in the AIH cohort (31.1% vs. 18.0%, *p* < 0.05). Fibrosis was also more severe in patients with NAFLD than in those without (53.8% vs. 39.9%, *p* < 0.05). Age, Plts, IgG and the presence with MADs were identified as independent predictors of the severity of inflammation in AIH patients. Moreover, severe liver fibrosis (stages 3 to 4) was independently associated with male (OR, 2.855; *p* = 0.025), γ-GT (OR, 0.997; *p* = 0.007), and combination with MADs (OR, 4.917; *p* = 0.006). Furthermore, combination with DM was also an independent predictor of severe liver fibrosis in AIH patients (OR, 2.445, *p* = 0.038).

**Conclusions:**

Concurrent MADs, common in AIH patients, is an independent risk factor for severe fibrosis or inflammation; of note, combination with DM was also an independent predictor of severe liver fibrosis in AIH patients. While managing with AIH, routine assessment of co-existing MADs, especially DM, is also important.

**Supplementary Information:**

The online version contains supplementary material available at 10.1007/s12072-024-10727-w.

## Introduction

Autoimmune hepatitis (AIH) is a chronic liver disease characterized by polyclonal hyperglobulinemia, autoantibodies, and interface hepatitis. AIH shows a favorable response to immunosuppressive treatment [1] and is observed in both sexes (30% males) [2] and any ethnic background [1]. Around 30% of AIH cases are reported in individuals over 60 years of age [1, 3]. On the other hand, metabolic syndrome is a group of metabolic abnormalities that include central obesity, type-2 diabetes mellitus (DM), hypertension (HT), and dyslipidemia [4, 5]. It is closely associated with an unhealthy lifestyle and insulin resistance [4]. Furthermore, metabolic syndrome contributes to the development of non-alcoholic fatty liver disease (NAFLD) [6], ranging from simple steatosis to non-alcoholic steatohepatitis (NASH), which can potentially progress toward hepatocellular carcinoma [7].

There is a close association between metabolic-associated diseases (MADs) (including obesity, DM, HT and dyslipidemia) and liver fibrosis in chronic hepatitis B (CHB) [8–10] or chronic hepatitis C (CHC) [11]. There is increased risk of all-cause mortality or malignancy from CHB patients with liver steatosis compared to those without steatosis [12].

In NAFLD, there is an association between liver fibrosis and metabolic syndrome [7, 13], implying MADs are independent risk factors of liver fibrosis [14]. Furthermore, the coincidence of AIH and NAFLD is an increased risk factor for the development of cirrhosis [15, 16]. Biochemical remission is less frequent at 3 years post-diagnosis among patients with one or more MADs from a US-based AIH study [17], showing that the AIH patients’ coincidence of MADs is less responsiveness to steroids. It is reasonable to hypothesize that such coincidence potentially increases risk of development of cirrhosis. Because liver fibrosis could progress into cirrhosis without proper management, it is a big challenge to clinicians to manage cirrhotic patients for the long-term survival prognosis of AIH patients [1].

However, it remains to be clarified if concurrent AIH and MADs increases risk to develop cirrhosis, as compared to those had AIH only. Thus, we determined that MADs, especially when combined with DM and/or NAFLD, are a risk factor for promoting hepatic fibrosis in AIH patients.

## Materials and methods

### Study population

We identified AIH patients who received liver biopsy at the Department of Infectious Diseases, Ruijin Hospital, Shanghai Jiaotong University, between January 2016 and February 2022. Liver biopsy was performed to clinically assess the severity of AIH. Exclusion criteria included age < 18 years, neoplasm (including hepatocellular carcinoma), other types of autoimmune liver disease (primary biliary cholangitis, primary sclerosing cholangitis, or autoimmune overlap syndrome), co-infection of hepatitis B and/or C, alcoholic liver disease (20 g/day or more for males, 10 g/day or more for females), drug- or toxin-induced liver disease, and heritage metabolic liver disease such as Wilson’s disease. To minimize potential intervention effects from the treatment, patients selected for the current study were naïve to immunosuppressive treatment. All procedures were approved by the Ethics Committee of Ruijin Hospital. The study was conducted in accordance with the Helsinki Declaration of 1975, as revised in 2008.

### Methods

In this retrospective study, we collected clinical parameters, laboratory data, imaging examination data, and biopsy reports from electronic medical records. Patients’ past medical histories were obtained from medical records. Clinical information was collected within 6 months of liver biopsy included age, sex, BMI, alcohol usage, and comorbid diagnoses such as HT, dyslipidemia, and DM. Overweight was defined as BMI ≥ 23 kg/m^2^, and obesity as BMI ≥ 25 kg/m^2^ [18–20]. DM, dyslipidemia and HT were defined according to reported medical history, specific treatment or diagnostic criteria[5, 17, 21]. Laboratory tests within 1 week of liver biopsy were recorded, including alanine transaminase (ALT), aspartate aminotransferase (AST), alkaline phosphatase (AKP), γ-glutamyl transferase (γ-GT), total bilirubin (TB), albumin (Alb), bile acid (BA), creatinine (Cr), triglyceride (TG), total cholesterol (TC), high-density lipoprotein (HDL), low-density lipoprotein (LDL), fasting plasma glucose (FPG), complete blood count, autoantibodies, IgG, and viral markers. Medication lists were reviewed for treatment of AIH, HT, dyslipidemia, and DM. The diagnosis criteria of NAFLD were as follows [22]: (1) diagnosed steatosis by biopsy of the liver or (2) imaging examination, such as CT, MRI, or ultrasound examinations, suggested NAFLD.

### Liver histopathology

Ultrasound-guided liver biopsy was performed as previously described [23], and independently evaluated by two pathologists in a blind fashion. The AIH diagnosis are based on of 2008 IAIHG simplified AIH score ≥ 6 [24]. Typical features of AIH include interface hepatitis, lymphocytic/lymphoplasmocytic infiltration in portal tracts, and hepatic rosette formation [24]. The histopathological assessment of the inflammatory grades and the fibrosis stages was based on the Metavir score [25]. Histologic findings were categorized based on the severity of fibrosis or inflammation. Stage ≥ 2 was defined as moderate liver fibrosis or inflammation, while stage ≥ 3 were defined as severe liver fibrosis or inflammation.

Currently, there is no established scoring system for assessing the activity of fatty liver in patients with AIH. For the purpose of analysis histopathological features, a robust score, “steatosis, activity, and fibrosis (SAF) score” established [26] and validated [27] in patients with NAFLD was used to evaluate steatosis, lobular inflammation, and ballooning degeneration. Using the SAF score, patients were classified into three groups—NASH, NAFL, and no NAFLD—according to the fatty liver inhibition of progression (FLIP) algorithm. All the liver specimens from eligible patients were retrospectively assessed for the SAF score and FLIP classification.

### Statistical analysis

Continuous variables were expressed as mean ± SD, and differences in continuous variables were examined for statistical significance using Student’s t test or Kruskal–Wallis rank-sum test depending on the distribution of the data. Categorical variables were presented as number (percentage), and differences in categorical variables were analyzed with the Chi-squared test or Fisher’s exact. Correlation analyses were performed according to Spearman’s method. Logistic regression analyses were used to identify risk factors associated with severe fibrosis or inflammation. All the variables with a *p* value < 0.1 in univariate analysis entered the stepwise selection process, and those with a *p* value < 0.05 were retained. Before the construction of the multivariable logistic regression for severe liver fibrosis or inflammation, variables were assessed through univariable logistic regression. Results of univariable analyses are shown in Table 3. TC was excluded from univariable logistic regression due to its strong correlation with HDL or LDL (Pearson correlation coefficient, 0.559 or 0.870; *p* < 0.001, respectively). PT was excluded from univariable logistic regression because of its strong correlation with TB, TC, LDL or AFP (all Pearson correlation coefficient > 0.4; *p* < 0.001, respectively). BA was excluded from univariable logistic regression due to its poor specificity. In addition, the variable “gender (male)”, which was not significant in the univariable analysis, was nevertheless included in the multivariable models because of its sociological relevance (see Table 3). The results were expressed as Odds ratios (ORs) and 95% confidence intervals (CIs). A *p* values < 0.05 were considered significant.

## Results

### Clinical characteristics of study population

**Fig. 1:**
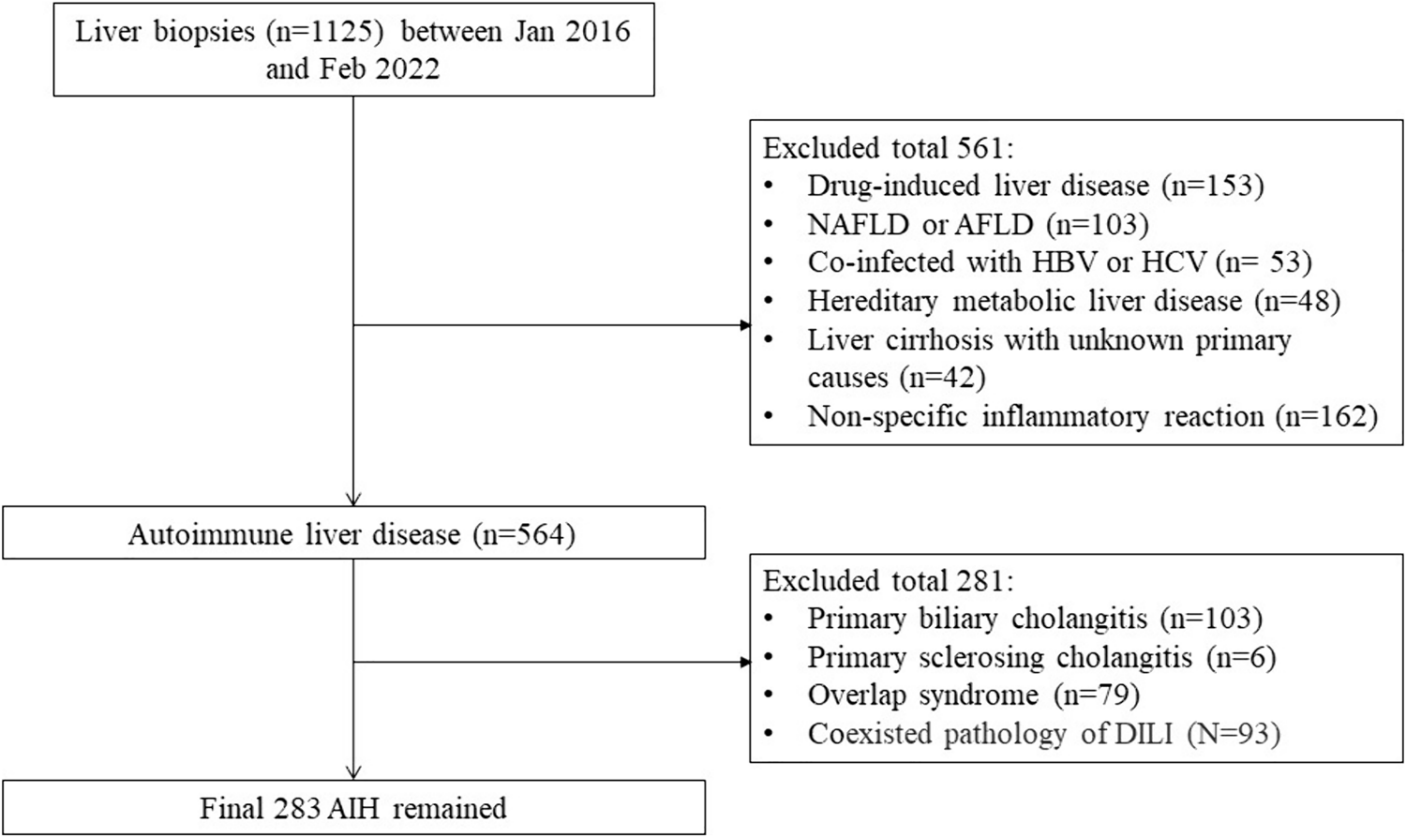
Inclusion and exclusion criteria for the AIH patients

A total of 283 AIH patients were included in the study, identified from 1125 patients who underwent liver biopsy at Ruijin Hospital (Fig. 1). The median age of the patients was 54.9 years, and the majority (82.3%) were females. Among the AIH patients, 75.9% were over 50 years old. ALT elevation was presented in 71.7% (203/283) of the population. Approximately 50.9% of AIH patients had moderate liver fibrosis, and 22.6% had moderate liver inflammation. Severe fibrosis (*S* ≥ 3) was observed in 43.1%, while severe inflammation (*H* ≥ 3) was observed in 77% of patients (Table 1).

**Table 1:** Lab characteristics in liver fibrosis or inflammation stage of AIH patients

Characteristic	Total *n* = 283	Fibrosis		*p* value	Inflammation		*p* value
		1–2 *n* = 161	3–4 *n* = 122		1–2 *n* = 65	3–4 *n* = 218	
Gender							
Male	50 (17.7%)	23 (14.3%)	27 (22.1%)	ns	7 (10.8%)	43 (19.7%)	ns
Female	233 (82.3%)	138 (85.7%)	95 (77.9%)		58 (89.25)	175 (80.3%)	
Age (Y)	54.9 ± 11.3	54.0 ± 10.3	56.1 ± 12.5	ns	51.7 ± 12.1	55.9 ± 10.9	< 0.01
≤ 29	9 (3.2%)	3 (1.9%)	6 (4.9%)	ns	4 (6.2%)	5 (2.3%)	ns
30–39	20 (7.1%)	13 (8.1%)	7 (5.7%)	ns	6 (9.2%)	14 (6.4%)	ns
40–49	39 (13.8%)	28 (17.4%)	11 (9.0%)	< 0.05	9 (13.8%)	30 (13.8%)	ns
50–59	103 (36.4%)	64 (39.8%)	39 (32.0%)	ns	26 (40.0%)	77 (35.3%)	ns
60–69	98 (34.6%)	47 (29.2%)	51 (41.8%)	< 0.05	19 (29.2%)	79 (36.2%)	ns
≥ 70	14 (4.9%)	6 (3.7%)	8 (6.6%)	ns	1 (1.5%)	13 (6.0%)	ns
BMI (kg/m^2^)	23.0 ± 3.3	23.0 ± 3.2	23.1 ± 3.5	ns	22.8 ± 3.3	23.1 ± 3.4	ns
< 23	153 (54.1%)	89 (55.3%)	64 (52.4%)	ns	39 (60.0%)	114 (52.3%)	ns
23–24.9	57 (20.1%)	26 (16.1%)	31 (25.4%)	ns	11 (16.9%)	46 (21.15)	ns
≥ 25	73 (25.8%)	46 (28.6%)	27 (22.1%)	ns	15 (23.1%)	58 (26.6%)	ns
ALT (IU/L)	113.3 ± 125.5	116.1 ± 121.7	109.7 ± 130.9	ns	84.4 ± 105.6	121.5 ± 129.7	< 0.05
AST (IU/L)	117.3 ± 131.3	119.7 ± 137.3	114.1 ± 123.4	ns	76.7 ± 92.8	128.8 ± 138.3	< 0.01
AKP (IU/L)	155.0 ± 99.1	160.5 ± 99.3	147.7 ± 98.9	ns	164.8 ± 111.8	152.3 ± 95.4	ns
γ-GT (IU/L)	173.6 ± 204.1	195.2 ± 236.6	144.7 ± 146.0	< 0.05	204.9 ± 271.0	164.7 ± 180.5	ns
TB (μmol/L)	32.8 ± 45.3	29.0 ± 32.4	37.9 ± 57.9	ns	23.6 ± 28.4	35.5 ± 48.7	ns
Alb (g/L)	38.4 ± 5.4	39.0 ± 5.0	37.5 ± 5.8	< 0.05	40.2 ± 4.5	37.8 ± 5.5	< 0.01
BA (μmol/L)	49.2 ± 59.3	41.4 ± 51.3	59.8 ± 67.3	< 0.05	30.9 ± 39.2	54.3 ± 62.8	< 0.01
Cr (μmol/L)	63.1 ± 16.0	62.1 ± 12.6	64.6 ± 19.8	ns	61.0 ± 11.5	63.7 ± 17.0	ns
Glu (mmol/L)	5.1 ± 0.8	5.0 ± 0.7	5.1 ± 0.9	ns	5.0 ± 0.7	5.1 ± 0.8	ns
TG (mmol/L)	1.6 ± 0.7	1.6 ± 0.6	1.7 ± 0.7	ns	1.5 ± 0.6	1.6 ± 0.7	ns
TC (mmol/L)	4.6 ± 1.6	4.7 ± 1.6	4.6 ± 1.5	ns	5.1 ± 1.4	4.5 ± 1.6	< 0.05
HDL (mmol/L)	1.2 ± 0.6	1.3 ± 0.6	1.2 ± 0.6	ns	1.4 ± 0.6	1.2 ± 0.6	< 0.05
LDL (mmol/L)	2.6 ± 1.1	2.6 ± 1.2	2.4 ± 1.1	ns	3.0 ± 1.0	2.4 ± 1.2	< 0.01
PT (s)	12.1 ± 1.3	11.8 ± 1.2	12.5 ± 1.3	< 0.001	11.5 ± 0.9	12.3 ± 1.3	< 0.001
WBC (× 10^9/L)	5.0 ± 1.6	4.9 ± 1.6	5.1 ± 1.6	ns	5.5 ± 1.9	4.8 ± 1.5	< 0.05
Plts (× 10^9/L)	171.3 ± 65.0	179.9 ± 66.3	159.9 ± 61.8	< 0.05	195.4 ± 70.3	164.2 ± 61.8	< 0.01
AFP (ng/mL)	13.4 ± 23.7	10.4 ± 21.1	17.3 ± 26.3	< 0.05	8.2 ± 21.2	14.7 ± 24.2	ns
ANA positivity	244 (86.2%)	132 (82.0%)	112 (91.8%)	< 0.05	50 (76.9%)	194 (89.0%)	< 0.05
IgG (mg/dL)	1704.4 ± 665.6	1609.3 ± 506.4	1829.9 ± 815.8	< 0.01	1494.8 ± 522.9	1766.9 ± 691.4	< 0.01
Normal	109 (40.1%)	67 (42.9%)	42 (36.2%)	< 0.01	27 (44.3%)	82 (38.9%)	ns
> 1 × UNL	39 (14.3%)	29 (18.6%)	10 (8.6%)		12 (19.7%)	27 (12.8%)	
> 1.1 × UNL	124 (45.6%)	60 (38.5%)	64 (55.2%)		22 (36.1%)	102 (48.3%)	
NAFLD							
With	65 (23.0%)	30 (18.6%)	35 (28.7%)	< 0.05	10 (15.4%)	55 (25.2%)	ns
Without	218 (77.0%)	131 (81.4%)	87 (71.3%)		55 (84.6%)	163 (74.8%)	

Data are presented as number (%) or mean ± SD

*AFP* alpha-fetoprotein, *Alb* albumin, *ALT* alanine aminotransferase, *AKP* alkaline phosphatase, *ANA* anti-nuclear antibody, *AST* aspartate aminotransferase, *BA* bile acid, *BMI* body mass index, *Cr* creatinine, *Glu* glucose, *γ-GT* γ-glutamyl transferase, *HDL* high density lipoprotein, *LDL* low density lipoprotein, *Plts* platelets, *PT* prothrombin time, *TB* total bilirubin, *TC* total cholesterol, *TG* triglyceride, *WBC* white blood cell

In AIH patients with severe liver fibrosis, there were higher levels of BA, PT, ANA positivity, IgG, or AFP and lower levels of γ-GT, Alb, or platelets (Plts) count (all *p* < 0.05) compared to patients with mild or moderate liver fibrosis. Similarly, in AIH patients with severe liver inflammation, there were higher levels of median age, ALT, AST, BA, PT, ANA positivity, or IgG and lower levels of Alb, WBC, Plts, TC, HDL, or LDL (all *p* < 0.05) compared to patients with mild or moderate liver inflammation (Table 1).

### Prevalence of MADs in AIH patients

**Fig. 2:**
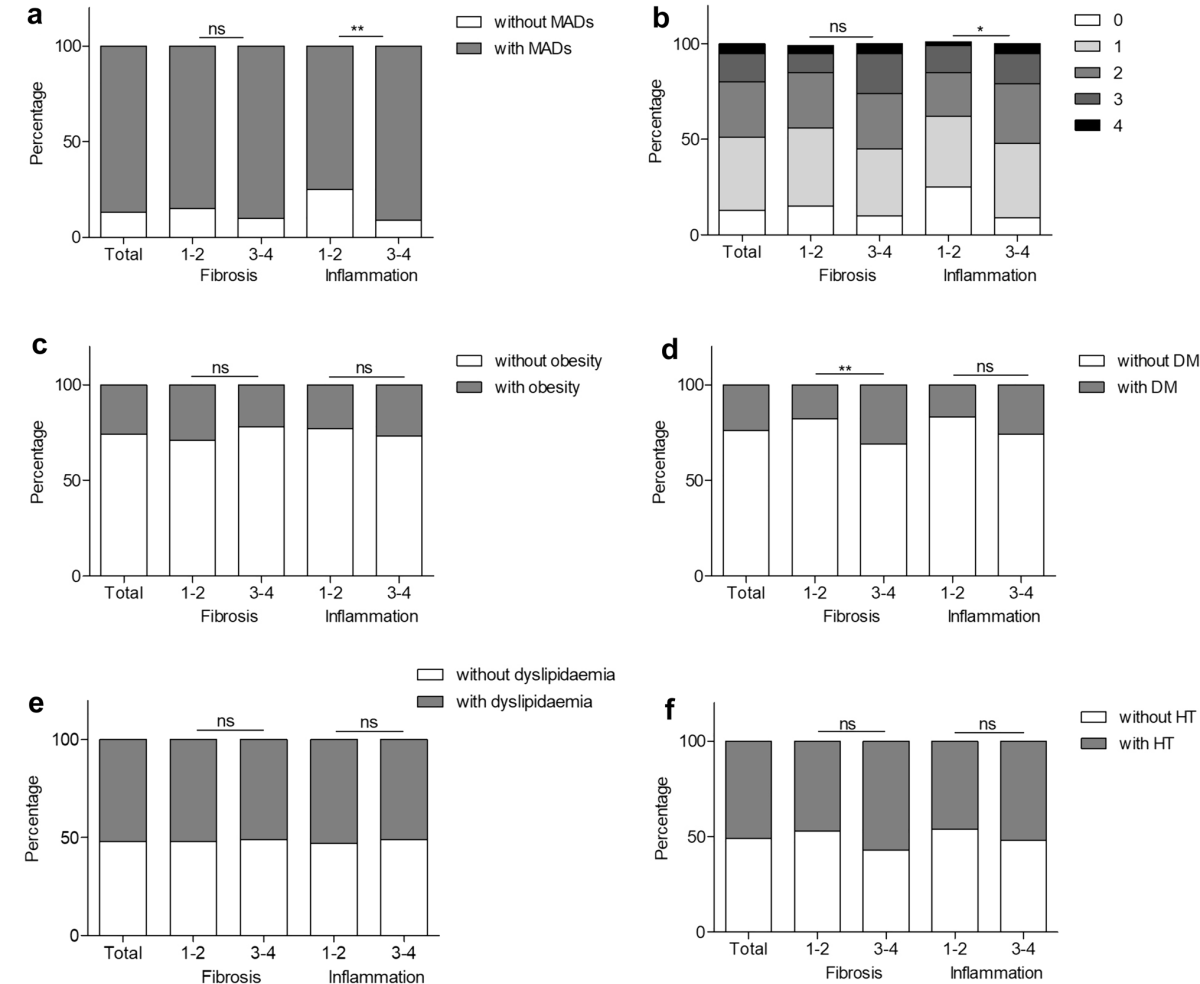
The proportion of MADs (**a**), number of MADs (**b**), obesity (**c**), DM (**d**), dyslipidaemia (**e**), HT (**f**) in different inflammation or fibrosis group of AIH patients

The prevalence of MADs in AIH patients was high (87.3%) (Fig. 2), with a large proportion of patients having multiple MADs. Specifically, 38.4%, 29.4%, 15.1%, or 4.5% of patients had 1, 2, 3, or 4 MADs, respectively. The overall prevalence of obesity, DM, HT, or dyslipidaemia was 25.8%, 23.7%, 50.9%, or 51.7%, respectively. DM was more prevalent in the severe fibrosis cohort compared to the mild or moderate fibrosis cohort, with AIH patients in the severe fibrosis cohort being 1.7 times more likely to have DM than those in the mild or moderate fibrosis cohort (31.1% vs. 18%, *p* < 0.05) (Fig. 2). Moreover, AIH patients in the severe inflammation group had a significantly higher proportion of MADs compared to those in the mild or moderate inflammation group, with the severe inflammation group having 1.2 times the prevalence of MADs compared to the mild or moderate inflammation group (90.7% vs. 75%, *p* < 0.05) (Fig. 2).

### Comparison in terms of the presence of NAFLD

**Fig. 3:**
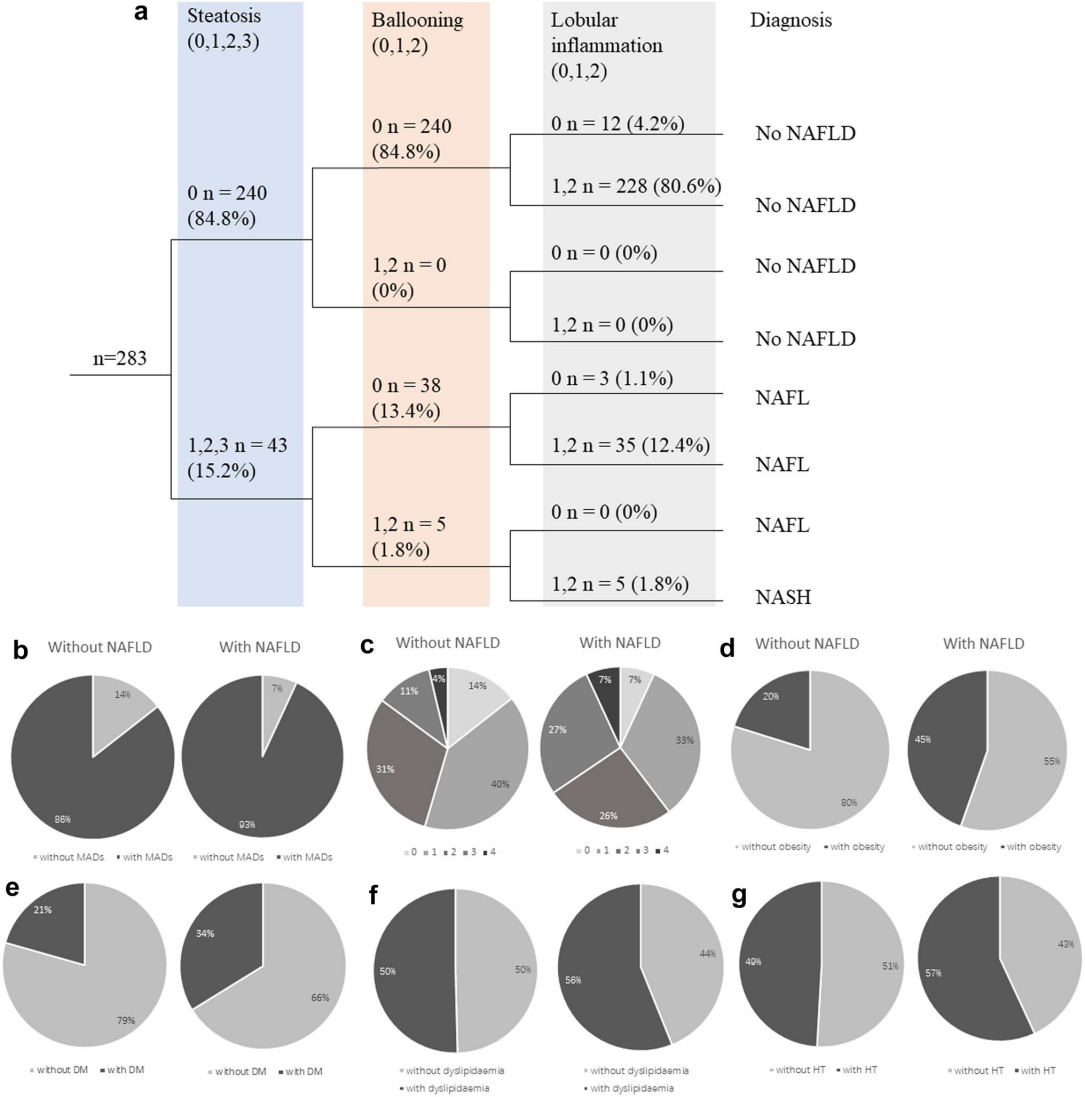
The FLIP algorithmic tree (**a**): a total of 283 AIH patients were classified into no NAFLD, NAFL, NASH, or severe NASH according to the FLIP algorithmic pathways based on the grade of steatosis (light blue shade), grade of ballooning (light orange shade), and grade of lobular inflammation (gray shade). Number of patients and the proportion among each category were presented as number (%). The proportion of MADs (**b**), number of MADs (**c**), obesity (**d**), DM (**e**), dyslipidaemia (**f**), HT (**g**) in the patients with or without NAFLD

The FLIP algorithmic tree shows the number of patients in the different algorithmic pathways (Fig. 3a), according to the liver biopsy diagnosis. Following the FLIP algorithm, 84.8% (240/283), 13.4% (38/283), and 1.8% (5/283) AIH patients were classified as without NAFLD, with NAFL, and with NASH, respectively.

In addition, from liver histopathology and imaging findings, the prevalence of NAFLD among the AIH patients was 23.0% (65/283), confirmed through either liver biopsy (43/65, 66.2%) or ultrasound/CT (22/65, 33.8%). The clinical characteristics of AIH patients were presented based on the presence or absence of NAFLD (Table 2). AIH patients with NAFLD had a mean age of 58.1 ± 9.6 years, a female percentage of 80.0%, and a mean BMI of 24.7 ± 3.6 kg/m^2^ (Table 2). There were significantly higher median age and BMI values (*p* < 0.05) in AIH patients with NAFLD than those without (Table 2). The age of AIH patients with NAFLD was significantly higher than those without at the age group 60–69 years, showing 50.8% vs. 29.8%; *p* < 0.05. Similarly, the proportion of AIH patients with BMI ≥ 25 in patients with NAFLD was also significantly higher than that in patients without NAFLD (44.6% vs. 20.2%, *p* < 0.05). Moreover, the proportion of AIH patients with NAFLD who developed severe fibrosis was significantly higher than in those without NAFLD (28.7% vs. 18.6%, *p* < 0.05) (Table 2).

**Table 2:** Lab characteristics at diagnosis in AIH patients ± NAFLD

Characteristic	Without NAFLD *n* = 218	With NAFLD *n* = 65	*p* value
Gender			
Male	37 (17.0%)	13 (20.0%)	ns
Female	181 (83.0%)	52 (80.0%)	
Age (Y)	54.0 ± 11.6	58.1 ± 9.6	0.009
≤ 29	9 (4.1%)	0 (0%)	ns
30–39	18 (8.3%)	2 (3.1%)	ns
40–49	30 (13.8%)	9 (13.8%)	ns
50–59	84 (38.5%)	19 (29.2%)	ns
60–69	65 (29.8%)	33 (50.8%)	< 0.05
≥ 70	12 (5.5%)	2 (3.1%)	ns
BMI (kg/m^2^)	22.5 ± 3.1	24.7 ± 3.6	0.000
< 23	132 (60.6%)	21 (32.3%)	< 0.05
23–24.9	42 (19.3%)	15 (23.1%)	ns
≥ 25	44 (20.2%)	29 (44.6%)	< 0.05
ALT (IU/L)	118.7 ± 126.7	95.5 ± 120.7	ns
AST (IU/L)	125.6 ± 139.0	89.6 ± 97.2	ns
AKP (IU/L)	161.7 ± 98.0	132.9 ± 100.4	0.042
γ-GT (IU/L)	184.7 ± 206.2	136.1 ± 193.9	ns
TB (μmol/L)	36.1 ± 50.4	22.0 ± 16.5	0.029
Alb (g/L)	37.8 ± 5.4	40.4 ± 5.1	0.000
BA (μmol/L)	53.3 ± 62.2	35.6 ± 45.9	0.038
Cr (μmol/L)	63.1 ± 15.2	63.4 ± 18.7	ns
Glu (mmol/L)	5.0 ± 0.7	5.4 ± 0.9	0.002
TG (mmol/L)	1.6 ± 0.7	1.8 ± 0.8	ns
TC (mmol/L)	4.6 ± 1.6	4.8 ± 1.6	ns
HDL (mmol/L)	1.2 ± 0.6	1.2 ± 0.4	ns
LDL (mmol/L)	2.4 ± 1.1	2.9 ± 1.1	0.011
PT (s)	12.1 ± 1.3	12.1 ± 1.4	ns
WBC (× 10^9^/L)	4.9 ± 1.7	5.4 ± 1.5	0.039
Plts (10^9^/L)	171.3 ± 68.6	171.2 ± 51.8	ns
AFP (ng/mL)	13.5 ± 22.9	13.1 ± 26.6	ns
ANA positivity	181 (85.8%)	57 (87.7%)	ns
ASMA positivity	5 (2.7%)	2 (3.9%)	ns
Anti-SLA positivity	7 (3.2%)	0 (0%)	ns
Anti-LKM-1 positivity	3 (1.6%)	2 (4.0%)	ns
Anti-LC-1 positivity	12 (6.3%)	1 (2.0%)	ns
IgG (mg/dL)	1728.5 ± 685.4	592.1	ns
Normal	88 (41.7%)	21 (34.4%)	ns
> 1 × UNL	25 (11.8%)	14 (23.0%)	
> 1.1 × UNL	98 (46.4%)	26 (42.6%)	
Liver fibrosis			
1	13 (6.0%)	4 (6.2%)	0.000
2	118 (54.1%)	26 (40.0%)	
3	68 (31.2%)	16 (24.6%)	
4	19 (8.7%)	19(29.2%)	
Liver inflammation			
1	1 (0.5%)	0	ns
2	54 (24.8%)	10 (15.4%)	
3	133 (61.0%)	44 (67.7%)	
4	30 (13.8%)	11 (16.9%)	

Data are presented as number (%) or mean ± SD

*AFP* alpha-fetoprotein, *Alb* albumin, *ALT* alanine aminotransferase, *AKP* alkaline phosphatase, *ANA* anti-nuclear antibody, *AST* aspartate aminotransferase, *BA* bile acid, *BMI* body mass index, *Cr* creatinine, *Glu* glucose, *γ-GT* γ-glutamyl transferase, *HDL* high density lipoprotein, *LDL* low density lipoprotein, *Plts* platelets, *PT* prothrombin time, *TB* total bilirubin, *TC* total cholesterol, *TG* triglyceride, *WBC* white blood cell, *Y* years

AIH patients with NAFLD exhibited a significantly higher proportion of MADs (93.1% vs. 85.6%, *p* < 0.01) (Fig. 3b). In more detailed, it was demonstrated that among patients with NAFLD, the proportion of patients with one, two, three, or four MADs was 32.8%, 25.9%, 27.6%, or 6.9%, respectively (Fig. 3c). Moreover, a correlation was observed between NAFLD and an increased frequency of obesity, and DM in AIH patients (Fig. 3d, e). However, there was no significant differences among other laboratory tests, (e.g., ALT, AST, γ-GT, Cr, ANA positivity and IgG) between AIH patients with and without NAFLD (Table 2).

### Factors associated with severity of liver fibrosis or inflammation

Spearman’s correlation analysis was conducted to investigate potential correlations between the presence of MADs or NAFLD and the incidence of severe fibrosis or inflammation, as well as other clinical factors (Supplement Table 1). There was a significant correlation between hepatic inflammation and male gender, age, ALT, AST, TB, Alb, BA, Cr, TC, LDL, WBC, Plts, PT, AFP, ANA positivity, IgG, the presence of MADs, DM, and number of MADs (Supplement Table 1). In addition, there was a significant correlation between the severity of fibrosis and age, AKP, γ-GT, TB, BA, Plts, PT, AFP, ANA positivity, IgG, and the presence of DM, HT or NAFLD (Supplement Table 1).

### Analysis of risk factors associated with severe liver fibrosis or inflammation

Using multivariable analysis, Age, Plts, IgG and the presence with MADs (Table 3) were identified as independent predictors of the severity of inflammation in AIH patients. Male gender, and γ-GT were identified as independent factors associated with severe fibrosis (Table 3). The presence of MADs was identified as an independent predictor of severe liver fibrosis in AIH patients (Odds ratio [OR], 4.917, *p* < 0.05), indicating at least a four-fold risk of developing severe liver fibrosis compared to the AIH patients without MADs. Furthermore, the presence of DM was identified as an independent predictor of severe liver fibrosis in AIH patients (OR, 2.445, *p* < 0.05), indicating more than a twofold increase risk of developing severe liver fibrosis.

**Table 3:** Univariable analyses and multivariable analyses of factor associated with severe inflammation or fibrosis in AIH patients

Variable	Severe inflammation (*H* ≥ 3)				Severe fibrosis (*S* ≥ 3)			
	Univariable analysis		Multivariable analysis		Univariable analysis		Multivariable analysis	
	OR (95% CI)	*p* value	OR (95% CI)	*p* value	OR (95% CI)	*p* value	OR (95% CI)	*p* value
Male	2.036 (0.868–4.774)	ns			1.705 (0.922–3.152)	ns	2.855 (1.142–7.139)	0.025
Age (Y)	1.031 (1.007–1.056)	0.010	1.042 (1.000–1.085)	0.047	1.016 (0.995–1.038)	ns		
Age (< 60 vs. ≥ 60)	1.643 (0.909–2.968)	ns			1.908 (1.176–3.096)	0.009		
BMI (kg/m^2^)	1.030 (0.946–1.121)	ns			1.013 (0.944–1.088)	ns		
ALT (IU/L)	1.003 (1.000–1.007)	0.045			1.000 (0.998–1.002)	ns		
AST (IU/L)	1.005 (1.001–1.009)	0.010			1.000 (0.998–1.002)	ns		
AKP (IU/L)	0.999 (0.996–1.001)	ns			0.999 (0.996–1.001)	ns		
γ-GT, IU/L	0.999 (0.998–1.000)	ns			0.999 (0.997–1.000)	0.049	0.997 (0.995–0.999)	0.007
TB (μmol/L)	1.015 (1.000–1.029)	0.047			1.005 (0.998–1.012)	ns		
Alb (g/L)	0.919 (0.869–0.972)	0.003			0.949 (0.907–0.993)	0.024		
BA (μmol/L)	1.009 (1.002–1.017)	0.010			1.005 (1.001–1.009)	0.013		
Cr (μmol/L)	1.013 (0.990–1.036)	ns			1.009 (0.993–1.026)	ns		
TG (mmol/L)	1.455 (0.841–2.517)	ns			1.190 (0.793–1.787)	ns		
TC (mmol/L)	0.813 (0.661–1.000)	0.050			0.959 (0.802–1.146)	ns		
HDL (mmol/L)	0.496 (0.281–0.877)	0.016			0.794 (0.492–1.280)	ns		
LDL (mmol/L)	0.649 (0.479–0.879)	0.005			0.859 (0.671–1.100)	ns		
PT (s)	1.865 (1.386–2.509)	0.000			1.638 (1.324–2.028)	0.000		
WBC (× 10^9^/L)	0.800 (0.679–0.944)	0.008			1.089 (0.942–1.259)	ns		
Plts (× 10^9^/L)	0.993 (0.989–0.997)	0.001	0.990 (0.984–0.996)	0.001	0.995 (0.991–0.999)	0.012		
AFP (ng/ml)	1.020 (0.996–1.045)	ns			1.013 (1.001–1.025)	0.037		
ANA positivity	2.425 (1.185–4.963)	0.015			2.461 (1.149–5.270)	0.020		
IgG (mg/dL)	1.001 (1.000–1.001)	0.004	1.002 (1.001–1.003)	0.022	1.001 (1.000–1.001)	0.007		
MADs (no vs. yes)	2.352 (1.105–5.006)	0.027	5.050 (1.433–17.789)	0.012	4.020 (1.694–9.542)	0.002	4.917 (1.578–15.320)	0.006
Number of MADs (< 2 vs. ≥ 2)	1.861 (1.003–3.454)	0.049			1.802 (1.090–2.978)	0.022		
Number of MADs (< 3 vs. ≥ 3)	1.585 (0.696–3.610)	ns			2.413 (1.286–4.527)	0.006		
Obesity (no vs. yes)	1.208 (0.630–2.316)	ns			0.711 (0.411–1.228)	ns		
DM (no vs. yes)	1.697 (0.829–3.472)	ns			2.059 (1.182–3.588)	0.011	2.445 (1.050–5.690)	0.038
Dyslipidaemia (no vs. yes)	1.114 (0.564–2.200)	ns			0.902 (0.517–1.574)	ns		
HT (no vs. yes)	1.279 (0.734–2.229)	ns			1.493 (0.930–2.396)	ns		
NAFLD (no vs. yes)	1.856 (0.886–3.889)	ns			1.757 (1.006–3.069)	0.048		

*AFP* alpha-fetoprotein, Alb albumin, ALT alanine aminotransferase, AKP alkaline phosphatase, ANA anti-nuclear antibody, *AST* aspartate aminotransferase, *BA* bile acid, *BMI* body mass index, *Cr* creatinine, *Glu* glucose, *γ-GT* γ-glutamyl transferase, *HDL* high density lipoprotein, *HPL* hyperlipidemia, *HT* hypertension, *LDL* low density lipoprotein, *MADs* metabolic-associated diseases, *NAFLD* alcoholic fatty liver disease, *Plts* platelets, *PT* prothrombin time, *TB* total bilirubin, *TC* total cholesterol, *TG* triglyceride, *WBC* white blood cell, *Y* years

## Discussion

In our current study, approximately 40% of AIH patients had severe liver fibrosis, with MADs present in 87.3% and NAFLD in 23.0% of AIH patients, respectively. In the severe fibrosis cohort, the proportion of AIH patients with DM was higher than that in the mild or moderate fibrosis cohort. Compared with AIH patients without NAFLD, those with NAFLD were older (median age), had higher BMI, and exhibited more severe fibrosis. Interestingly, only male, γ-GT, the presence with MADs and the presence with DM were identified as independent factors associated with severe fibrosis, using multivariable analysis.

The precise underlying mechanism of AIH remains to be explored, but it is speculated that it is due to autoimmune-mediated attacks against hepatocytes with unknown cause(s) [1]. AIH commonly affects middle-aged women [28], as supported by our current study, which showed that the median age of all patients was 54.9 years and the majority were female (82.3%). In addition, we found that approximately 94% of AIH patients had at least moderate liver fibrosis at the time of definitive diagnosis by liver biopsy, and around half of them presenting with severe fibrosis.

In our cohort, we found that the prevalence of NAFLD was 23.0%, but other study has shown a prevalence of 17% [16]. Interestingly, we observed a significant difference in median age between AIH patients with and without NAFLD. Our study utilized a combination of liver histology, ultrasound and CT to detect hepatic steatosis, which may explain the higher prevalence of NAFLD compared to comorbidity rate based solely on liver histopathology [16]. Diagnosing AIH with NAFLD can be challenging because some patients with ANA-positive NAFLD may be misdiagnosed, leading to delayed diagnosis and treatment. In addition, liver biopsy may have certain limitations due to potential sampling errors. Therefore, to diagnose NAFLD in our study, we utilized a variety of examination methods, including pathology, ultrasound, and CT. This approach may have increased the likelihood of detecting NAFLD in AIH patients. Our findings of older age in AIH patients with NAFLD are consistent with previous studies that have identified older age as a characteristic of AIH patients with concurrent NAFLD. Moreover, the distribution of both AIH and NAFLD in adults shows a single peak in patients in their 60s [28, 29], which further supports our observations of older age in AIH patients with NAFLD.

Unhealthy lifestyle habits, such as irregular eating and low physical activity[4], contribute to obesity [6] and are a major cause of the increased prevalence of MADs worldwide. In our study, we observed that AIH patients with NAFLD had higher BMI and a higher proportion of obesity than patients without NAFLD, consistent with previous research linking metabolic syndrome and NAFLD [29]. Previous studies have demonstrated a close association between liver fibrosis and metabolic syndrome in NAFLD [30], with MADs being independent risk factors for liver fibrosis [14]. In our study, we reported a high prevalence of MADs in AIH patients. Although there was no significant difference in the prevalence of MADs between severe and mild/moderate liver fibrosis, we found that the combination of MADs could be an independent factor for severe fibrosis in AIH patients in multivariate analysis. This finding aligns with previous research demonstrating an association between MADs and liver fibrosis in other chronic liver diseases [8]. In addition, we attempted to determine which single factor might be more important in promoting liver fibrosis, further analysis demonstrated that DM is also an independent risk factor. This supports the concept that MADs promote liver fibrosis, and aligns with previous study in other chronic liver diseases, showing that the presence of DM in CHB patients is associated with an increased risk of liver fibrosis, and cirrhosis occurrence [31]. In our study group, the prevalence of MADs in AIH patients was high, and 75.9% of patients were over 50 years old. We have primarily described patients diagnosed with AIH, who, in addition to being older, also have a significant proportion of comorbid metabolic-associated diseases. Therefore, we aim to investigate whether these comorbid conditions affect inflammation and fibrosis in AIH patients. However, this does not allow us to infer that patients with metabolic-associated diseases are at risk for AIH, as the study population differs. We did not assess the incidence of AIH among patients with metabolic-associated diseases, but this is an important research point. In future studies, we could explore whether the prevalence of AIH varies among specific patient populations.

In our cohort, fibrosis and/or cirrhosis is a common feature when a high proportion of patients are diagnosed with AIH. Therefore, it appears to be impossible to distinguish fibrosis caused by AIH or MADs, given the high comorbidity rate of MADs. However, MADs, including hypertension, diabetes, dyslipidemia, and obesity, do not themselves constitute causes of cirrhosis. Cirrhosis, as an outcome of end-stage chronic liver disease, is more commonly associated with metabolic disorders causing NAFLD, which can serve as a foundation for chronic liver disease leading to cirrhosis. In our current retrospective cohort study, relatively few of AIH patients had NAFLD. In addition, our retrospective study primarily focused on patients with AIH to explore the impact of concurrent MADs on inflammation or fibrosis in AIH patients. Due to the specific characteristics of patient medical history data in liver disease at the time, we were unable to collect sufficient data on risk factors related to MADs, such as high-sugar or high-salt diets. However, we plan to address this issue in future studies.

Previous research reports that AIH patients with NAFLD often exhibit mild elevations in hepatobiliary enzymes compared to those without NAFLD [16]. However, we did not find a significant difference in hepatobiliary enzymes between AIH patients with and without NAFLD. Such discrepancy could be attributed to the high degree of liver fibrosis in our cohort, leading to a presentation of chronic hepatitis rather than acute hepatitis. In addition, we observed a significantly higher percentage of patients with severe fibrosis in the NAFLD subgroup than in the non-NAFLD subgroup, which may have contributed to the lack of difference in liver-enzyme indicators between the two groups.

In addition, we found that AIH patients with NAFLD had a higher percentage of severe fibrosis and were more likely to present with cirrhosis. This is consistent with previous research, demonstrating that the coincidence of AIH and NAFLD is more likely to lead to cirrhosis [15]. However, concurrent NAFLD was not identified as an independent risk factor for severe liver fibrosis or liver inflammation in univariate analysis. This finding is consistent with previous research, which found that hepatic steatosis is not associated with liver fibrosis in AIH patients, using non-invasive vibration-controlled transient elastography [32]. However, this may also be due to the relatively small sample size. NAFLD from the current retrospective study was not identified as an independent factor for severe liver fibrosis using restricted multivariable statistical analysis. We will verify this issue in future studies from multiple centers with different genetic as well as environmental backgrounds.

Most cases of AIH present with one or more significant titers of autoantibodies. ANA, smooth muscle antibodies (SMA), and antibodies to kidney microsome-1 (anti-LKM1) are standard autoantibodies. According to the pattern of autoantibodies detected, AIH is classified into two types: 1. ANA and/or SMA antibodies characterize type 1 AIH (AIH-1), which accounts for almost 90% of cases; 2. anti-LKM1 and antibodies to liver cytosol type 1 (anti-LC1) characterize type-2 AIH (AIH-2) [33]. In our current study, the positivity for anti-SMA was very low, i.e., 2.7%, which is much lower than the recommended diagnostic criteria for AIH, where positivity for anti-SMA is in the range of 80% [34]. The reasons may be that some practitioners focus more on ANA antibodies during initial screening, possibly overlooking SMA. In fact, upon reviewing the original data from our current study, the detection rate for ANA was 100%, while for SMA it was only around 84%. Approximately 46 patients lacked SMA detection data, which might have contributed to the lower observed positivity rate in statistics. In addition, all these participating patients were confirmed as AIH based on comprehensive scoring systems, including autoantibodies and typical AIH liver histopathological features in our current retrospective study. Some patients were still diagnosed with AIH based on pathology, IgG levels, and overall scoring, even though ANA antibodies were negative. Thus, we believe that the AIH diagnosis is reliable. The positivity rates of ANA and SMA varied among different populations with AIH in different disease states, which is supported by a previous study on AIH-related acute liver failure patients in the US, where the positivity rate for ANA was 70% and for SMA was 43% [35]. Therefore, different populations may have varying rates of autoantibody positivity. Given that our study is retrospective with a relatively small sample size and liver biopsy being an invasive procedure, patients' consent for liver biopsy may contribute to selection bias to some degree. We will minimize the selection-bias in future studies by including multi-center data from different genetic and environmental backgrounds.

We acknowledge the limitations of our current study. First, the sample size was relatively small, and the cross-sectional study design limits our ability to establish a firm causal relationship. Second, we did not investigate the correlation between MADs or NAFLD and prognosis, which will be explored in future studies. Third, we chose NAFLD, rather than metabolic-associated fatty liver disease (MAFLD), as one of MADs exposure in the study. Because of the concept of MAFLD was defined in 2020 [36], while our current retrospective study focused on the period from 2016 to 2020. Due to lacked data on abdominal circumference, we are unable to test the concept of MAFLD in the current study, but it will be determined in future study.

In summary, our study provides novel insights suggesting that MADs, particularly when combined with DM, are significant risk factors for promoting hepatic fibrosis in AIH patients. These findings underscore the importance of considering and managing metabolic factors in the management of AIH.

## Supplementary Information

Below is the link to the electronic supplementary material.

Supplementary file1 (DOCX 33 KB)

## Data Availability

All data from manuscript will be available upon request by contacting Dr Hui Wang.

## Abbreviations

AFP: Alpha-fetoprotein
AIH: Autoimmune hepatitis
Alb: Albumin
ALT: Alanine aminotransferase
AKP: Alkaline phosphatase
ANA: Anti-nuclear antibody
AST: Aspartate aminotransferase
BA: Bile acid
BMI: Body mass index
CHB: Chronic hepatitis B
CHC: Chronic hepatitis C
Cr: Creatinine
DM: Diabetes mellitus
Glu: Glucose
γ-GT: γ-Glutamyl transferase
HDL: High density lipoprotein
HT: Hypertension
LDL: Low density lipoprotein
MADs: Metabolic-associated diseases
MAFLD: Metabolic-associated fatty liver disease
NAFLD: Non-alcoholic fatty liver disease
OR: Odds ratio
Plts: Platelets
PSL: Prednisolone
PT: Prothrombin time
TB: Total bilirubin
TC: Total cholesterol
TG: Triglyceride
WBC: White blood cell
