# Supplementary material for: Liver fibrosis is closely linked with metabolic-associated diseases in patients with autoimmune hepatitis
Source: Hepatol Int. 2024 Sep 9;18(5):1528–39. doi: 10.1007/s12072-024-10727-w (PMC11461548; doi:10.1007/s12072-024-10727-w)
Supplement: Supplementary file 1 — Supplementary file1 (DOCX 33 KB) [file 12072_2024_10727_MOESM1_ESM.docx]

Supplement Table 1. Spearman’s correlation analysis of factors associated with liver inflammation or fibrosis.

| Parameters | Liver Inflammation | | Liver Fibrosis | |
| --- | --- | --- | --- | --- |
|  | r_s_ | p-value | r_s_ | p-value |
| Gender (male *vs* female) | -0.155 | < 0.01 | -0.084 | ns |
| Age (Y) | 0.167 | < 0.01 | 0.194 | < 0.01 |
| BMI (kg/m^2^) | 0.089 | ns | 0.086 | ns |
| ALT (IU/L) | 0.153 | < 0.05 | -0.037 | ns |
| AST (IU/L) | 0.234 | < 0.001 | 0.021 | ns |
| AKP (IU/L) | 0.012 | ns | -0.142 | < 0.05 |
| γ-GT (IU/L) | -0.022 | ns | -0.130 | < 0.05 |
| TB (μmol/L) | 0.251 | < 0.001 | 0.220 | < 0.001 |
| Alb (g/L) | -0.242 | < 0.001 | -0.115 | ns |
| [BA](javascript:;) (μmol/L) | 0.279 | < 0.001 | 0.160 | < 0.01 |
| Cr (μmol/L) | 0.127 | < 0.05 | 0.037 | ns |
| Glu (mmol/L) | 0.084 | ns | 0.066 | ns |
| TG (mmol/L) | 0.004 | ns | 0.037 | ns |
| TC (mmol/L) | -0.171 | < 0.05 | -0.064 | ns |
| HDL (mmol/L) | -0.109 | ns | -0.045 | ns |
| LDL (mmol/L) | -0.196 | < 0.01 | -0.704 | ns |
| PT (s) | 0.370 | < 0.001 | 0.373 | < 0.001 |
| WBC (×10^9/L) | -0.118 | < 0.05 | 0.047 | ns |
| Plts (×10^9/L) | -0.231 | < 0.001 | -0.228 | < 0.001 |
| AFP (ng/ml) | 0.283 | < 0.001 | 0.278 | < 0.001 |
| ANA positivity | 0.195 | < 0.01 | 0.146 | < 0.05 |
| IgG (mg/dl) | 0.227 | < 0.001 | 0.142 | < 0.05 |
| MADs | 0.177 | < 0.01 | 0.114 | ns |
| Number of MADs | 0.177 | < 0.01 | 0.114 | ns |
| Obesity (no *vs* yes) | 0.093 | ns | 0.002 | ns |
| DM (no *vs* yes) | 0.170 | < 0.01 | 0.165 | < 0.01 |
| Dyslipidaemia (no *vs* yes) | -0.044 | ns | -0.024 | ns |
| HT (no *vs* yes) | 0.062 | ns | 0.122 | < 0.05 |
| NAFLD (no *vs* yes) | 0.092 | ns | 0.159 | < 0.01 |

AFP, alpha-fetoprotein; Alb, albumin; ALT, alanine aminotransferase; AKP, alkaline phosphatase; ANA, anti-nuclear antibody; AST, aspartate aminotransferase; BA, [bile acid](javascript:;); BMI, body mass index; Cr, creatinine; DM, diabetes mellitus; Glu, glucose; γ-GT, γ-glutamyl transferase; HDL, high density lipoprotein; HPL, hyperlipidemia; HT, hypertension; LDL, low density lipoprotein; MADs, metabolic associated diseases; NAFLD, alcoholic fatty liver disease; Plts, Platelets; PT, prothrombin time; TB, total bilirubin; TC, total cholesterol; TG, triglyceride; WBC, white blood cell.
